# Effectiveness of Fish Oil-DHA Supplementation for Cognitive Function in Thai Children: A Randomized, Doubled-Blind, Two-Dose, Placebo-Controlled Clinical Trial

**DOI:** 10.3390/foods11172595

**Published:** 2022-08-26

**Authors:** Phakkharawat Sittiprapaporn, Akkarach Bumrungpert, Prayoon Suyajai, Con Stough

**Affiliations:** 1 Neuropsychological Research Laboratory, Department of Anti-Aging and Regenerative Science, School of Anti-Aging and Regenerative Medicine, Mae Fah Luang University, Bangkok 10110, Thailand; 2 Research Center of Nutraceuticals and Natural Products for Health & Anti-Aging, College of Integrative Medicine, Dhurakij Pundit University, Bangkok 10210, Thailand; 3 Department of Psychology, Faculty of Humanities, Mahachulalongkornrajavidyalaya University, Phra Nakhon Si Ayutthaya 13170, Thailand; 4 Center for Human Psychopharmacology, Swinburne University, P.O. Box 218 Hawthorn, Melbourne 3122, Australia

**Keywords:** brain, cognition, memory, fish oils, fatty acid, omega-3

## Abstract

The effects of fish oil (FO) or omega-3 supplementation on cognition has been the subject of several previous clinical trials. However, the effect of different doses taken chronically on cognition in children has not been well studied. In order to address this gap in our knowledge, we conducted a randomized, double-blind, placebo-controlled clinical trial. A total of one hundred and twenty healthy, cognitively normal Thai children aged 6–12 years old consumed daily low dose FO (260 mg Docosahexaenoic acid (DHA)), high dose FO (520 mg DHA), or placebo (Soybean oil) for 12 weeks. Cognitive function was assessed using a computerized cognitive battery, including the Go/NoGo, N-Back, and Digit Span tests as well as concurrent event-related potentials (ERPs), which together measured attention, processing speed, inhibition, and memory at baseline and 12 weeks. We hypothesized that compared to placebo, the two FO groups would show improved cognitive performance and shorter ERP latencies. In total, 42, 39, and 39 participants completed each of the test (FO-A, FO-B) and placebo groups (P) allocations, respectively, and were analyzed (120 in total across the three groups). No significant differences were observed between reaction times (RTs), accuracy, or error rates for all three of the cognitive tests. The ERP measurement and analysis of brain activity during the cognitive tests showed an increase in ERP amplitude. For all cognitive tests, there was a dose-response effect of FO on ERP amplitudes. These findings indicate that fish oil intake leads to a consistent improvement in attention and cognitive processing ability measured by changes in brain activity during working and long-term memory processes. This is the first study to directly quantify such an effect through simultaneous measurement of manual and mental activity during cognitive tasks following chronic FO use in children.

## 1. Introduction

Fish oil contains omega-3 (n-3) long-chain polyunsaturated fatty acids (LC-PUFA). Eicosapentaenoic acid (EPA) and Docosahexaenoic acid (DHA) are required for brain formation and function [1]. Dietary intake of EPA and DHA is often reflected in blood levels using the omega-3 index. In epidemiologic studies, a lower omega-3 index has been linked to a range of poorer outcomes, including overall death, ischemic stroke, decreased brain volume, impaired cognition, and mental illnesses, amongst other conditions [1]. Omega-3 fatty acids are essential dietary fatty acids, playing important roles in neural, visual, immune, cardiovascular, integumentary system, and connective tissue functions. DHA and EPA are especially critical in the developing brain and retina. DHA has a more distinct role in brain and eye function, whilst EPA has greater links to cardiovascular function, inflammation, and immunity. This creates a demand for DHA and EPA and increases the risk of health issues, especially cognitive and visual deficits [1].

DHA is an essential omega-3 PUFA that must be taken through diet [2]. In mammalian grey matter, DHA is the most abundant omega-3 PUFA. In the frontal brain of adult humans, PUFAs account for roughly 15–20% of total fat composition [2,3]. During childhood, it accumulates by means of placental nutrient transfer from the mother during pregnancy and then via breast milk, or supplemental sources, after birth. A dietary gap may occur when children transition to eating solids [2]. Dietary sources of DHA, for example, salmon and mackerel, are not usually found on the plates of children and may lead to suboptimal levels of DHA absorption [2,3,4]. DHA is implicated in autonomic functions, attention, and inhibition, all of which are components of executive function, such as working memory, planning, and mental plasticity, which are all related to frontal lobe development [2]. Previous investigations have revealed that DHA intake is related to neurocognitive development, particularly on measures of attention and memory [2,3,4,5]. The DHA-rich frontal cortex is responsible for executive function and higher-order psychological activities, including planning, critical thinking, problem-solving, and focused attention [6]. Haag demonstrated relationships between omega-3 PUFA levels in the central nervous system and its activity during psycho-pathological situations [7]. Similarly, DHA assists in the development of cognitive ability in healthy children [2,3,4,5,6,7,8], despite contrary evidence where low-level DHA for up to 6 months was shown to have no effect on working memory or cognitive plasticity compared to a placebo [9].

Cognition includes several functions, including memory, general intelligence, learning, language, orientation, perception, attention, concentration, and judgment, which are collectively regarded as important markers in a child’s development and academic achievement [10,11]. A child’s ability to think, reason, and employ executive function serves a critical role in daily and social behaviors. The development of this capacity is reported to occur throughout childhood and adolescence and is mirrored by structural and functional changes that occur in the brain throughout this time [12]. Cognitive ability throughout childhood and adolescence is under the influence of a multitude of factors, including diet [13].

Several previous studies have reported the effects of FO supplementation on cognitive function, but few have included real-time measurement of brain function by measuring the electrical activity of the brain. Non-invasive, functional assessment of brain activity, including electronic measurement of event-related potentials (ERPs), provides valuable insights into the spatial and real-time activity of the brain during cognitive testing. The positive waveforms measured around 300 msec after a stimulus (P300) represents a well-characterized cognitive endogenous measure of attention and memory [14]. P300 voltage peaks in response to visual or auditory stimuli are detectable in infants and appear to represent identical cognitive processes to adults. However, the latency of the peak amplitude is greatly delayed at younger ages, and there are many more measurement artifacts, a possible reason why there are so few studies using ERPs after FO supplementation in children. P300-like components in infancy, voltage changes in atypically developing groups, P300-behavioral correlations, individual P300 variations, and P300 brain foundations throughout development are some of the main variables that can interfere with ERP analysis, and it is important to resolve these to understand the developmental dynamics of brain activity [15].

Since cognition is an important marker in a child’s development and academic achievement, the development of a child’s ability to think, reason, and employ executive functions are critical processes to measure throughout this time [10,11,12]. Several previous studies have reported the effects of FO supplementation on cognitive function, but few have included real-time measurement of brain function by measuring the electrical activity using the non-invasive, functional assessment of brain activity. ERPs provide valuable insights into the spatial and real-time activity of the brain during cognitive testing, and it is important to assess these to understand the developmental dynamics of brain activity [15]. Therefore, the present study evaluated the effects of FO supplementation on cognitive function processing ability, together with measures of working memory, in children aged between 6 and 12 years, following daily intake for 12 weeks. We employed ERP analysis of brain activity during standardized tests for memory, attention, and inhibition to provide a comprehensive picture of real-time brain responses to standard cognitive testing. Electronic measurement of ERPs also provides valuable insights into the spatial and real-time activity of the brain during cognitive testing in order to demonstrate a change in brain activity during cognitive test performance in children due to daily FO supplementation after 12 weeks. The ERP measures work concurrently with the cognitive measures and may be considered to be more sensitive than the cognitive measures, given their measurement is in milliseconds as opposed to seconds. Given the inconsistencies in previous research studies with FO supplementation outlined earlier, it is plausible that a more sensitive marker of cognitive improvement measured at the millisecond level may be important in understanding changes in brain function after FO supplementation. Therefore, we hypothesized that FO supplementation would improve the processing speed measured by ERPs during a demanding cognitive test. A differentiation between the behavioral (cognitive) tasks and the ERP outcomes may, to some extent, explain differences in the previous literature on whether FO supplementation improves cognitive and brain function in children. This is an important study because of the wide use of FO supplementation in children and the assumption that FO supplementation improves cognitive and brain function. This needs to be specifically addressed by well-controlled studies.

## 2. Materials and Methods

### 2.1. Study Design

**Figure 1:**
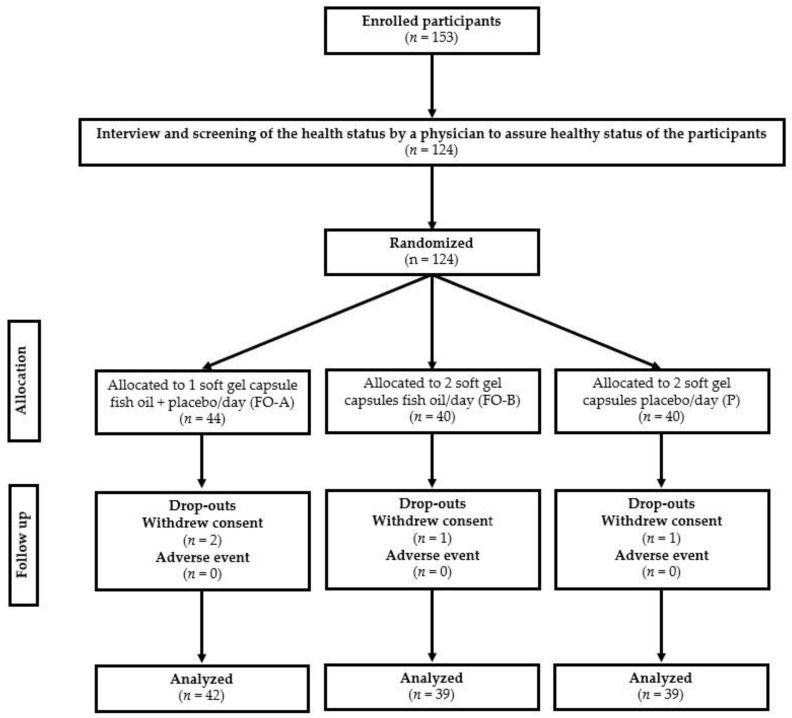
Schematic diagram showing intervention allocation groupings in the study.

**Figure 2:**
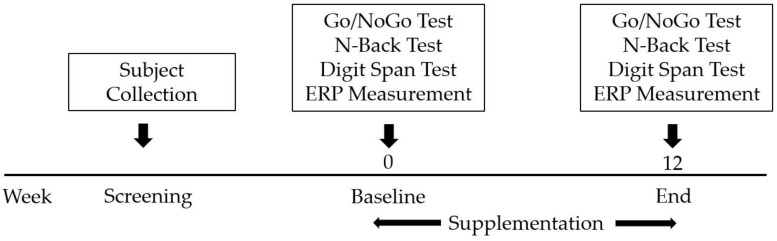
The timeline of cognitive battery task performance and event-related potential (ERP) assessments.

We used a randomized, placebo-controlled, double-blind, parallel-group design for this study. Eligible participants were recruited from March to October 2020 until the target sample size was achieved. Participants were generally healthy, and no serious medical conditions were reported by the parents of the recruits. After enrolment, participants were assigned identification numbers (ID) in ascending order and randomly allocated to one of three allocations: (1) one capsule of FO plus one capsule of placebo/day; (2) two chewable capsules of FO/day; (3) or a placebo, using a blocked, randomized list, until equal proportions per group were reached. The study sponsor labeled all investigational products with participant IDs before study commencement, and the study site team received a blinded list of participant IDs. Participants and investigators were blinded to the treatment allocation. Participants were instructed to consume two chewable capsules of their allocated investigational material once a day at the same time in the morning throughout the 12 weeks study period. Participants attended a total of two study visits (baseline and week 12). The cognitive function of each participant was determined using the computerized battery test at baseline level (prior to supplement consumption) and at 12 weeks (after completion of the course of investigational product consumption). The protocol flow chart followed in the study is summarized in Figure 1 and Figure 2.

### 2.2. Participants

Parents or legal guardians of all the children who participated in the study gave informed consent prior to enrollment. Study inclusion criteria were: 6–12 years old, no continuous consumption (i.e., regular or everyday consumption) of fish oil in the preceding three months. Individuals with self-reported chronic, malignant disease (e.g., cancer, heart, liver, renal, or other metabolic diseases), psychiatric or neurological diseases, prior head injury, and FO allergy were excluded. Enrolled participants visited the study site at screening, week 0 (baseline), and 12th week. Demographic details, including educational background, gender, and age of the children, were collected (Table 1). All participants used Thai as the primary language, and their health conditions were confirmed via physical examination by the project physician. The Institutional Ethics Committee, Mahachulalongkornrajavidyalaya University, Thailand, gave their approval to the study (Code: 121/2021), and it was carried out in conformity with the ethical criteria set forth in the 1964 Helsinki Declaration and its subsequent revisions. Four persons withdrew due to inconvenient transportation: two from FO-A, one from FO-B, and one from P groups, respectively.

### 2.3. Investigational Product

The FO supplement used in this study was a commercially available preparation. Both FO and placebo products were provided by Max Biocare, Pty. Ltd (manufactured by Catalent Inc., Aprilia, Italy) traded as BrightKids^®^ (also marketed as Dasbrain^®^). The product is high-quality, pharmaceutical grade tuna oil sourced from Iceland Lysi h.g and provides omega-3 fatty acids—DHA and EPA—in a chewable, soft capsule format. Enriched for DHA, it is designed to support brain and eye development and function and is also indicated to support immunity and cardiovascular and musculoskeletal health and is suitable for children and adults. The product is manufactured in a certified Good Manufacturing Practice (GMP) licensed facility in Australia, a member of the International Council for Harmonisation of Technical Requirements for Pharmaceuticals for Human Use (ICH) and the Pharmaceutical Inspection Convention (PIC/S). Ingredients are from clean, high-quality sources, and the product is free from added egg, dairy, yeast, peanut, gluten, artificial colors, or preservatives. The active composition of omega-3 fatty acids is shown in Table 2. Since the product only contained purified DHA and EPA as the active ingredients, any biological effects of FO over the placebo are attributed to these omega-3s. The placebo itself (260 mg soybean oil) was formulated to have the same excipients, flavor, odor, appearance, and texture as the investigation FO product. The soyabean oil contained 7% omega-3 fatty acids (as alpha-linolenic acid) and 87% omega-6 fatty acids (primarily oleic, linoleic, and palmitic acid) but no detectable EPA or DHA. All participants were requested to maintain their normal eating and exercise habits. Compliance was defined as missed consumption of the investigational product for three or more consecutive days.

### 2.4. Assessment of Cognitive Function

Cognitive function was measured both by ERP analysis via the oddball visual paradigm, and by the cognitive battery test of psychometric and psychological tests via a computer interface. Responses times were based on manual keyboard or mouse entry, and brain ERP detection was performed simultaneously.

#### 2.4.1. Cognitive Battery Testing

The cognitive battery of psychometric and psychological tests used in this study comprised 3 tasks, including Go/NoGo, N-Back, and Digit Span Forward tests. They were used in this study to monitor updating, shifting, and inhibition capacities of working memory. Administration of the assessments to the participants was by research assistants trained and/or degree-holders in educational psychology and blinded to the treatment assignment. The accuracy and response times during each test session were recorded and expressed in milliseconds and percentage accuracy.

(1) Go/NoGo Test. A modified version of the Go/No-Go task was performed by the participants [16]. Participants were exposed to a set of fish and shark images, and participants were asked to memorize the fish. According to this test, a list of fish and sharks was presented to the participant sequentially, and the participant was requested to memorize the fish and shark. The participant was required to recall them when they were asked to select only fish, but not sharks. During this task, fish and sharks that served as Go and No-Go signals were presented on a monitor at around 150 cm from the participant’s eyes. The fish represented the ‘Go’ condition with 80 percent probability, and the shark represented the ‘NoGo’ condition with 20 percent probability. The reaction buttons were placed under the participants’ palms in a soundproofed and electrically protected chamber. Participants had to press the response pad as quickly as they could (with their dominant hand) every time the more frequent fish (Go) stimulus appeared on the computer screen and withhold their reactions to the less frequent shark (NoGo) stimulus. The order of conditions was counterbalanced across participants.

(2) N-Back Test: Three sets of bear pictures were presented to the participant. According to this test, a list of moving bear positions was presented to the participant one at a time, for example, at 75°, 90°, and 180°. Participant was requested to memorize only the bear at the 90° position as it moved around. The participant was required to recall this target bear when it returned to the same place. During this task, the bear at the 90° position that served as the N-Back signal was presented on a monitor at around 150 cm from the participant’s eyes. The bear at 90° position represented the target N-Back condition with 20 percent probability, and the other positions represented the non-target condition with 20 percent probability. The reaction interface and order of conditions were similar to that used in the Go/NoGo test.

(3) Digit Span Test: Digit Span Forward is a measure of short-term memory capacity [17]. In the Forward task, a sequence of visual digits was played on the computer screen, and participants were asked to recall the random digit sequence in order [18]. A series of three random numbers printed in three different colors were presented to the participant. After that, four series of three random numbers printed in three different colors were presented. The participant was requested to press “ENTER” when the presented sequence of numbers matched. Only the Digit Span Forward task was administered.

#### 2.4.2. Event-Related Potential (ERP) Recording Procedure

ERP recordings were based on signals detected through the scalp with a wearable, multi-electrode array cap (Electro-cap, eego^TM^, ANT Neuro, PE Hengelo, The Netherlands). A 32-channel set of electrodes (Fp1, Fpz, Fp2, F7, F3, Fz, F4, F8, FC5, FC1, FC2, FC6, T7, C3, Cz, C4, T8, CP5, CPz, CP1, CP2, CP6, P7, P3, Pz, P4, P8, O1, O2, Oz, M1, M2) was pre-mounted within the elastic Electro-Cap (Waveguard^TM^ original EEG cap, ANT Neuro, PE Hengelo, The Netherlands) according to the International 10–20 Electrode Positioning System. The Waveguard™ EEG cap (ANT Neuro, PE Hengelo, The Netherlands) is easy to implement, using very thin electrode wires, and the flexible, breathing cap fabric enables comfortable recordings even over a longer period. Between the electrodes Fz and Cz, a ground electrode was attached. M1 and M2 reference electrodes were placed on ipsilateral mastoids, with Fp1 and Fp2 electrodes employed for ocular artifact detection. The resistance of the electrodes was less than 10 kΩ. With a 0.05 to 100 Hz band pass, the EEG signals were amplified, captured at 500 Hz, and live signal data were saved to a hard disk for offline processing. A 0.1–30 Hz band pass was then used to digitally filter recorded ERPs. The epoch on which the average was calculated as 500 milliseconds, and the baseline was 100 milliseconds before the commencement of the presenting stimuli. All neural and ocular artifacts were removed from the continuous EEG prior to extraction of ERP waves. The epochs were extracted from the EEG-free artifact from 100 msec pre-stimulus and continued to 500 msec post-stimulus. The baseline correction was also applied to each epoch, with any changes of voltage below 0.1 μV or above 70 μV rejected from further analysis.

After registration, the data were re-referenced offline to the common average montage, followed by correction and rejection of artifacts. EEG epochs with absolute amplitudes greater than 100 volts were automatically flagged and removed from further investigation. Before averaging, all channels were subjected to artifact rejection with a threshold of ±100 µV. ERP waveforms were generated to investigate the ERP components where the target stimuli evoked reactions of frequent stimuli, presenting with 80% probability. Infrequent stimuli for non-target conditions were presented randomly with a probability of 20% (oddball paradigm). The interstimulus interval was 1000 msec. The amplitude (μV) and latency (ms) of the ERP signals were measured. The total recording time was 5 min for each of the three cognitive tests. The positive peak that presented between 250 and 400 msec was defined as P300. Both latencies and amplitudes of both brainwaves were recorded and analyzed. All ERP analyses were performed using ASA^TM^ 4.0 analysis software (ANT Neuro, PE Hengelo, The Netherlands), featuring source reconstruction, signal analysis, and MRI processing tools.

The global field power (GFP) peak metric was used to assess the electric strength (hilliness) of a brain electric field map independent of its spatial layout. The spatial standard deviation of all voltage estimations was based on one spontaneous EEG map. A steep potential map would have a higher GFP peak than a flat potential map. The GFP described above was self-contained [19,20]. The spatial standard deviation of GFP quantifies the amount of activity at each time point in the field, resulting in a reference-independent descriptor of the potential field. The occurrence times of GFP maxima were used to determine the latencies of visually-evoked potential components, which became complimentary over time [21]. This was performed by averaging the ERPs from all scalp channels while excluding electrooculographic channels. Participants’ mean and grand mean GFP peak amplitudes were computed [19,20,21,22] and statistically analyzed as per the cognitive function tests.

### 2.5. Behavioral Recording and Analyses

Participants’ RTs and response accuracies and times were measured from their key presses during each cognitive function task. All cognitive function tasks were a two-force-choice experiment; thus, button presses were classified as correct responses (button code matched stimulus type); incorrect responses (button code did not match stimulus type), or missed (no button press). RTs for correct, incorrect, and missed responses were measured as the difference in timing between the stimulus onset and key press reaction. Only trials that had RTs between 100–1500 ms were included. This was performed to remove inadvertent button pressing and extremely delayed button pressing that might have resulted from distraction or cognitive fatigue. Reaction times were averaged and corrected for learning effects over time across trials for each participant.

### 2.6. Outcome Measurements

The primary outcome measurements were cognitive function tests, including Go/NoGo, N-Back, and Digit Span Tests, after the 12th week of synbiotic administration. The secondary outcome measurements were the ERP analysis of brain activity during standardized tests for memory, attention, and inhibition to provide a comprehensive picture of real-time brain responses to standard behavioral testing.

### 2.7. Statistical Analysis

Quantitative data are provided as means with standard deviations. The data were analyzed using SPSS Program (IBM) version 21.0, Renewal Quote Number: 26500879 (Mae Fah Luang University, Chiang Rai, Thailand). In order to determine the effects of fish oil supplementation on cognitive function over the time periods (baseline and 12th week), grand mean of GFP peak amplitudes, mean response times (RTs), and correct responses, as well as cognitive function analyses, were performed using an unpaired Student’s *t*-test. One-way ANOVAs were performed on accuracy and reaction times for the cognitive function tests. Tukey’s post hoc analyses were performed on significant ANOVA results. Statistical results were considered significant at *p* < 0.05 [22].

### 2.8. Ethics and Trial Registration

This study was performed according to the Declaration of Helsinki (ethical principles for research involving human subjects). All subjects’ parents gave informed consent for their children to participate in the trial. The trial protocol was approved by the Institutional Human Ethical Committee (ID No. 201/2021, 9 April 2020) of Mahachulalongkornrajavidyalaya University, Thailand. The clinical trial protocol was also registered with the Thai Clinical Trials Registry (TCTR20190418001) and the WHO International Clinical Trials Registry Platform (WHO-ICTRP) database.

## 3. Results

### 3.1. Participant Demographics

In total, 42, 39, and 39 participants completed each of the test (FO-A, FO-B) and placebo groups (PC) allocations, respectively, and were analyzed (120 in total across the 3 groups). The study dropout rate was low (*n* = 4, 0.32%), comprising FO-A (*n* = 2), FO-B (*n* = 1) and PC (*n* = 1) groups, respectively (Figure 1) and all participants were not compliant to the treatment schedule. Baseline characteristics, including demographics of participants in each group of the per-protocol (completed) sets, are presented in Table 2. FO-A, FO-B, and PC groups were generally well-balanced on baseline characteristics. There were more female participants overall compared to males (68 vs. 52), and the education of the participants was primary school level. There was no statistically significant difference in any of the baseline demographic characteristics between the groups.

### 3.2. Behavioral Effects of Fish Oil

The primary analysis was conducted on 120 participants in the FO-A, FO-B, and P (per-protocol) groups who completed the study. Means at baseline and at study completion for all cognitive function tests are reported in Table 3. No significant differences were observed in accuracy or error rates between the start and end of the behaviors testing.

#### 3.2.1. Go/NoGo

For the RTs from the Go/NoGo task, the two-way (treatment x visit) interaction effect was not significant, with F (5,17) = 1.381, *p* = 0.298, indicating that the two-way interaction effects between treatments and visits were not different across groups.

#### 3.2.2. N-Back

For the RTs from the N-Back task, the two-way (treatment x visit) interaction effect was not significant, with F (5,17) = 0.39, *p* = 0.846, indicating that the two-way interaction effects between treatments and visits were not different across groups.

#### 3.2.3. Digit Span

For the RTs from the Digit Span Forward task, the two-way (treatment x visit) interaction effect was not significant, with F (5,17) = 0.271, *p* = 0.920, indicating that the two-way interaction effects between treatments and visits were not different across groups.

### 3.3. Effects of Fish Oil on Brain Activity

Table 4 shows the effect of FO supplementation on cognitive function assessed by the EEG analysis at baseline and at week 12. GFP is plotted over time, and the occurrence times of GFP maxima are used to determine the latencies of evoked potential components. The grand mean GFP peak amplitude and latency of the ERPs are shown for each of the cognitive function tests in Table 4.

#### 3.3.1. Go/NoGo

For Go/NoGo task mean amplitudes, the two-way (treatment x visit) interaction effect was significant, with F (5,17) = 19.91, *p* < 0.001. A comparison of the mean amplitudes of the FO-A, FO-B, and placebo groups was analyzed by the ANOVA model. The FO group outperformed the placebo by amplitude—5.64 (±0.85) for FO-A, 6.95 (±0.23) for FO-B, and 3.51 (±0.70) for P—on the 12th week. For Go/NoGo task latencies, the two-way (treatment x visit) interaction effect was significant, with F (5,17) = 3.232, *p* = 0.0445. The mean RTs of the FO-A, FO-B, and placebo groups from the ANOVA model. FO group outperformed the placebo by RT’s 479.35 (±6.82) msec for FO-A, 456.35 (±10.38) msec for FO-B, and 476.66 (±2.79) msec for P on the 12th week.

#### 3.3.2. N-Back

In N-Back task mean amplitudes, the two-way (treatment x visit) interaction effect was significant, with F (5,17) = 28.85, *p* < 0.001. The mean amplitudes for the FO-A, FO-B, and placebo groups were analyzed by the ANOVA model. The FO group outperformed placebo by amplitude 5.62 (±1.17) for FO-A, 7.71 (±0.30) for FO-B, and 3.31 (±0.59) for P at 12th week, but not for latency (F (5,17) = 0.5389, *p* = 0.7436).

#### 3.3.3. Digit Span

In Digit Span Forward task mean amplitudes, the two-way (treatment x visit) interaction effect was significant, with F (5,17) = 18.458, *p* < 0.0001. The mean amplitudes of the FO-A, FO-B, and placebo groups were analyzed by the ANOVA model. The FO group outperformed the placebo by an amplitude of 5.42 (±1.09) for FO-A, 7.22 (±0.53) for FO-B, and 3.53 (±0.79) for P at 12 weeks. For Digit Span Forward task latency, the two-way (treatment x visit) interaction effect was also significant, with F (5,17) = 3.192, *p* = 0.0462.

### 3.4. Adverse Events

No adverse event was reported during the study. All participants were able to continue consuming both FO and P until the end of the study.

## 4. Discussion

The two-way (treatment x visit) interaction effect was not significant for all of the cognitive function tests administered. However, there were some significant FO-related effects observed during ERP measurement. These results showed that the FO-B mean amplitude was significantly greater than either FP-A and P groups amplitude during all cognitive function tests. The RT of the FO-B group was shorter compared to either FO-A and P groups. We did not see any changes in the behavioral tests because our behavioral tests might not be sensitive enough to pick up changes in performance, whereas brain activity may be more sensitive (and measured more directly) to FO supplementation.

The linear regression analysis of a previous study showed that supplementing healthy school-aged children for 6 months with 300 mg/d DHA did not improve executive processes such as working memory and cognitive flexibility [9]. However, several research studies have revealed that DHA is essential for autonomic function, inhibition, and attention which are crucial parts of executive function. Working memory, mental flexibility, and planning are all dependent on frontal brain development [2,3,6,23]. Planned behavior and cognitive skills, including problem solving and creative thinking, have been proposed to be facilitated by DHA-rich frontal lobes [6]. Similar to short-term and working memory, FO showed an enhancing effect on selective and sustained attention indexed by brain electrical activities in our present study. One possible explanation is that attention comprises several semi-independent sub-systems, including selective attention, sustained attention, attentional switching, auditory-verbal working memory, or divided attention [24,25], and only some of these are probably improved by FO supplementation. In addition, several previous studies have suggested that omega-3 supplementation (EPA and DHA) for 35 days improved attentional and physiological processes, especially complex cortical processing [2,3,4,5,6,7,8]. However, the finding of our current investigation found that twelve weeks of fish oil supplementation improved responding time. RTs were faster in the 12th week after FO consumption compared to baseline RTs. Therefore, the result of our present study indicates a reduction in processing speed in the behavioral tests, which is consistent with previously reported studies [2,3,4,5,6,7,8] and consistent with the changes in ERP amplitudes that we observed.

A recent meta-analysis conducted by Jiao et al. [24] found that in 1031 infants, omega-3 supplementation significantly improved cognitive development, including psychomotor function, motor skills, and language. An earlier study conducted on 44 infants ranging in age from birth to 4 months found that supplementation with long-chain polyunsaturated fatty acids (metabolized to DHA) resulted in better problem-solving skills and more intentional solutions than those infants that were not supplemented [26]. In older children (6–10 years of age), the CHAMPION study (*n* = 645; a double-blind, controlled trial) revealed the addition of 100 mg of DHA in multiple micronutrient treatment regimes significantly improved cognitive performance (short-term memory and reasoning) [27]. Another clinical study demonstrated that administration of 4 g of fish oil daily (800 mg DHA and 1600 mg EPA) for 35 days significantly improved attention and reaction time and reduced the error rate during the attention test (versus placebo) in adult subjects aged 22-51 [28]. The same study also showed increased activity of the cortex [8].

It has been reported that omega-3 supplementation for one month improves cognition and neural efficiency in young adults (22–34 years old) [28]. DHA forms an important component of neuronal cells that allows them to grow and develop connections with other neurons. In isolated hippocampal neurons, for instance, exposure to DHA resulted in an increased number of neuron projections called neurites [25,29]. Neurite length, the number of branches on each neuron, the number of synapses (nerve cell junctions), and synaptic transmission are all significantly increased after exposure to DHA [25]. These findings suggest DHA supplementation assists the development of neuronal networks, in turn supporting normal brain development and cognitive functioning.

In addition, ERP changes are commonly associated with brain growth in children and adolescents who are in good health. Previous studies have examined changes in N2 and P3 components in patients with attention-deficit/hyperactivity disorder’s as they mature. In a recent study, researchers examined age-related variations in the auditory NoGo-N2 component in attention-deficit/hyperactivity disorder (ADHD) patients. The NoGo-N2 delay at Fz (6.08 msec) and Cz (4.88 msec) electrodes dropped, respectively, with a 1-year rise in age [30]. In those with ADHD, there were age-related variations in NoGo-N2 latency at the Fz and Cz electrodes [30].

By analyzing the P3 component, Ali et al. studied the topographic voltage distribution and post-attentive integration in dyslexic children. P3 amplitudes at the T4 electrode in the dyslexia group were much higher than in the control group. P300 voltage distribution was higher in the dyslexia group than the control group in the right parietal and left occipital areas. Children with dyslexia had a higher rate of post-attentive integration, and this process involved the parietal and occipital areas [31].

Although fish oil has been touted as having a number of benefits, it also can cause a few side effects, especially with high doses, including unpleasant taste, bad breath, smelly sweat, headache, heartburn, nausea and gastrointestinal discomfort, and diarrhea. Some people have reported headaches as a side effect of taking fish oil, but studies have also shown that omega-3s can be a headache reliever. In fact, taking a fish oil supplement is often a recommendation for people who suffer from chronic migraines [32]. The most common side effect of fish oil consumption is diarrhea [32]. Taking it with meals will help to curb this side effect. However, no serious adverse event was reported during the study. All participants were able to continue consuming both FO and P until the end of the study.

Our study is limited in the following ways: first, the relatively short 12-week study duration does not elucidate the long-term effects of fish oil consumption. Secondly, physiological measurements such as serum cortisol or other biochemistry markers were not measured. Thus, though an effect was demonstrated, changes in physiological parameters and their correlation with observed effects that may explain mechanisms of action were not investigated in this study. As our study has shown heterogeneity in terms of the effect of FO on cognitive and brain function, other physiological and biochemical measurements should also be measured in order to elucidate mechanisms of action. This randomized, double-blind, placebo-controlled clinical trial demonstrated that fish oil consumption in healthy children improved working memory, assessed by brain electrical activities during behavioral tests after daily consumption for twelve weeks.

## 5. Conclusions

This study is the first study to demonstrate a change in brain activity during cognitive test performance due to FO supplementation. The current data did not show any cognitive-enhancing effect of fish oil on RTs in the Go/NoGo, N-Back, and Digit Span Forward behavioral tests, while there was a commensurate increase in ERP amplitude, suggesting that there are direct subtle changes in brain activity rather than changes in cognitive test performance. Fish oil supplementation for 12 weeks duration in children improves the processing of information in the brain during tasks of cognitive function.

## Figures and Tables

**Table 1:** The demographic data of participants.

Demographic and Baseline Characteristics	FO-A (*n* = 42)	FO-B (*n* = 39)	P (*n* = 39)	*p*-Value
Age (years), mean (SD)	9.45 (±1.88)	10.18 (±1.47)	9.74 (±1.50)	0.14
Male	16	16	20	-
Female	26	23	19	-
Education				
1st grade	12	-	4	-
2nd grade	3	8	2	-
3rd grade	1	6	11	-
4th grade	14	4	13	-
5th grade	4	13	1	-
6th grade	8	8	8	-

FO-A: low dose fish oil (1 × fish oil, 260 mg DHA, with 1 × placebo—Soybean oil capsule); FO-B: high dose fish oil (2 × capsules of fish oil, 520 mg DHA); P: placebo group (2 × 520 mg Soybean oil capsules).

**Table 2:** Nutritional content of study investigational products.

Content	FO-A	FO-B	Placebo
Docosahexaenoic acid (DHA)	260 mg	520 mg	-
Eicosapentanoeic acid (EPA)	60 mg	120 mg	-
Total omega-3 triglycerides	320 mg	640 mg	36 mg *

* as C18:3 alpha-linolenic acid.

**Table 3:** The effect of fish oil (FO) on the cognitive function battery test, compared to placebo group.

Cognitive Function Test	Groups	Mean (SD) Score at Baseline	Mean (SD) Score at 12th Week
**Go/NoGo**			
(%) Accuracy (Go)	FO-A (*n* = 42)	94.72	96.11
	FO-B (*n* = 39)	98.50	99.19
	P (*n* = 39)	96.92	98.33
(%) Error (Go)	FO-A (*n* = 42)	5.28	3.89
	FO-B (*n* = 39)	1.50	0.81
	P (*n* = 39)	3.08	1.67
Reaction time (ms) (Go)	FO-A (*n* = 42)	601.39 (±10.63)	601.46 (±31.21)
	FO-B (*n* = 39)	563.23 (±40.65)	534.94 (±20.70)
	P (*n* = 39)	580.60 (±66.90)	575.90 (±24.49)
(%) Accuracy (NoGo)	FO-A (*n* = 42)	85.24	91.55
	FO-B (*n* = 39)	86.28	89.87
	P (*n* = 39)	87.18	87.56
(%) Error (NoGo)	FO-A (*n* = 42)	14.76	8.45
	FO-B (*n* = 39)	13.72	10.13
	P (*n* = 39)	12.82	12.44
**N-Back**			
(%) Accuracy	FO-A (*n* = 42)	90.20	94.72
	FO-B (*n* = 39)	90.73	95.13
	P (*n* = 39)	92.44	95.21
(%) Error	FO-A (*n* = 42)	9.80	5.28
	FO-B (*n* = 39)	9.27	4.87
	P (*n* = 39)	7.56	4.79
Reaction time (ms)	FO-A (*n* = 42)	479.69 (±50.879)	466.33 (±55.154)
	FO-B (*n* = 39)	462.20 (±38.190)	452.98 (±93.199)
	P (*n* = 39)	428.33 (±95.728)	418.83 (±16.502)
**Digit Span**			
(%) Accuracy	FO-A (*n* = 42)	70.08	72.22
	FO-B (*n* = 39)	81.20	76.32
	P (*n* = 39)	74.53	78.59
(%) Error	FO-A (*n* = 42)	29.92	27.78
	FO-B (*n* = 39)	18.80	23.68
	P (*n* = 39)	25.47	21.41
Reaction time (ms)	FO-A (*n* = 42)	1063.71 (±129.87)	1124.75 (±85.827)
	FO-B (*n* = 39)	1240.0 (±519.10)	1121.27 (±53.455)
	P (*n* = 39)	1039.9 (±156.67)	1133.71 (±13.806)

FO-A: One chewable soft gel capsule of fish oil (260 mg DHA) plus one chewable soft gel capsule of placebo (260 mg Soybean oil) (*n* = 42); FO-B: Two chewable soft gel capsules of fish oil (520 mg DHA) (*n* = 39); P: Two chewable soft gel capsules of placebo (520 mg Soybean oil) (*n* = 39).

**Table 4:** The effect of fish oil (FO) on the grand mean global field power (GFP) of event-related potentials (ERPs) component.

Cognitive Function Test	Groups	Mean (SD) Scores at Baseline	Mean (SD) Scores at 12th week
**Go/NoGo**			
Latency (ms)	FO-A (*n* = 42)	477.68 (±9.81)	479.35 (±6.82)
	FO-B (*n* = 39)	474.66 (±5.18)	456.35 (±10.38)
	P (*n* = 39)	476.66 (±11.12)	476.66 (±2.79)
Amplitude (*µ*V)	FO-A (*n* = 42)	3.69 (±0.91)	5.64 (±0.85) *
	FO-B (*n* = 39)	2.16 (±0.20)	6.95 (±0.23) ***
	P (*n* = 39)	2.90 (±0.90)	3.51 (±0.70)
**N-Back**			
Latency (ms)	FO-A (*n* = 42)	462.20 (±14.49)	455.17 (±7.81) *
	FO-B (*n* = 39)	463.44 (±9.10)	455.33 (±15.44) ***
	P (*n* = 39)	466.95 (±4.45)	467.75 (±19.83)
Amplitude (*µ*V)	FO-A (*n* = 42)	2.93 (±0.86)	5.62 (±1.17) *
	FO-B (*n* = 39)	2.12 (±0.55)	7.71 (±0.30) ***
	P (*n* = 39)	2.70 (±0.47)	3.31 (±0.59)
**Digit Span**			
Latency (ms)	FO-A (*n* = 42)	457.59 (±9.91)	454.42 (±7.99)
	FO-B (*n* = 39)	467.45 (±0.46)	453.12 (±10.54)
	P (*n* = 39)	464.94 (±10.37)	444.91 (±1.76)
Amplitude (*µ*V)	FO-A (*n* = 42)	3.40 (±0.56)	5.42 (±1.09) **
	FO-B (*n* = 39)	2.52 (±0.67)	7.22 (±0.53) ***
	P (*n* = 39)	2.85 (±0.58)	3.53 (±0.79)

FO-A: One chewable soft gel capsule of fish oil (260 mg DHA) plus one chewable soft gel capsule of placebo (260 mg Soybean oil) (*n* = 42); FO-B: Two chewable soft gel capsules of fish oil (520 mg DHA) (*n* = 39); P: Two chewable soft gel capsules of placebo (520 mg Soybean oil) (*n* = 39); * *p* value < 0.05, ** *p* < 0.01, and *** *p* < 0.001, respectively, compared to the placebo group.

## Data Availability

The data presented in this study are available on request from the corresponding author.
